# Supplementary figures and images for: Framework for analyzing MAE-derived immunopeptidomes from cell lines with shared HLA haplotypes
Source: PLoS One. 2025 Sep 25;20(9):e0332950. doi: 10.1371/journal.pone.0332950 (PMC12463239; doi:10.1371/journal.pone.0332950)

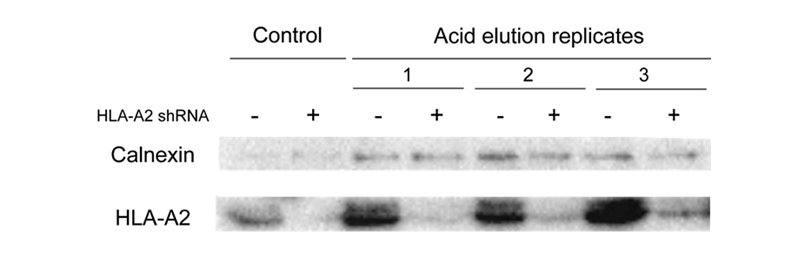

Supplement: S1 Fig — shRNA knockdown of HLA-A2 in the cell line GM2709 was performed and compared against a control knockdown. Blot against calnexin served as a loading control. (TIF) [file pone.0332950.s001.tif]

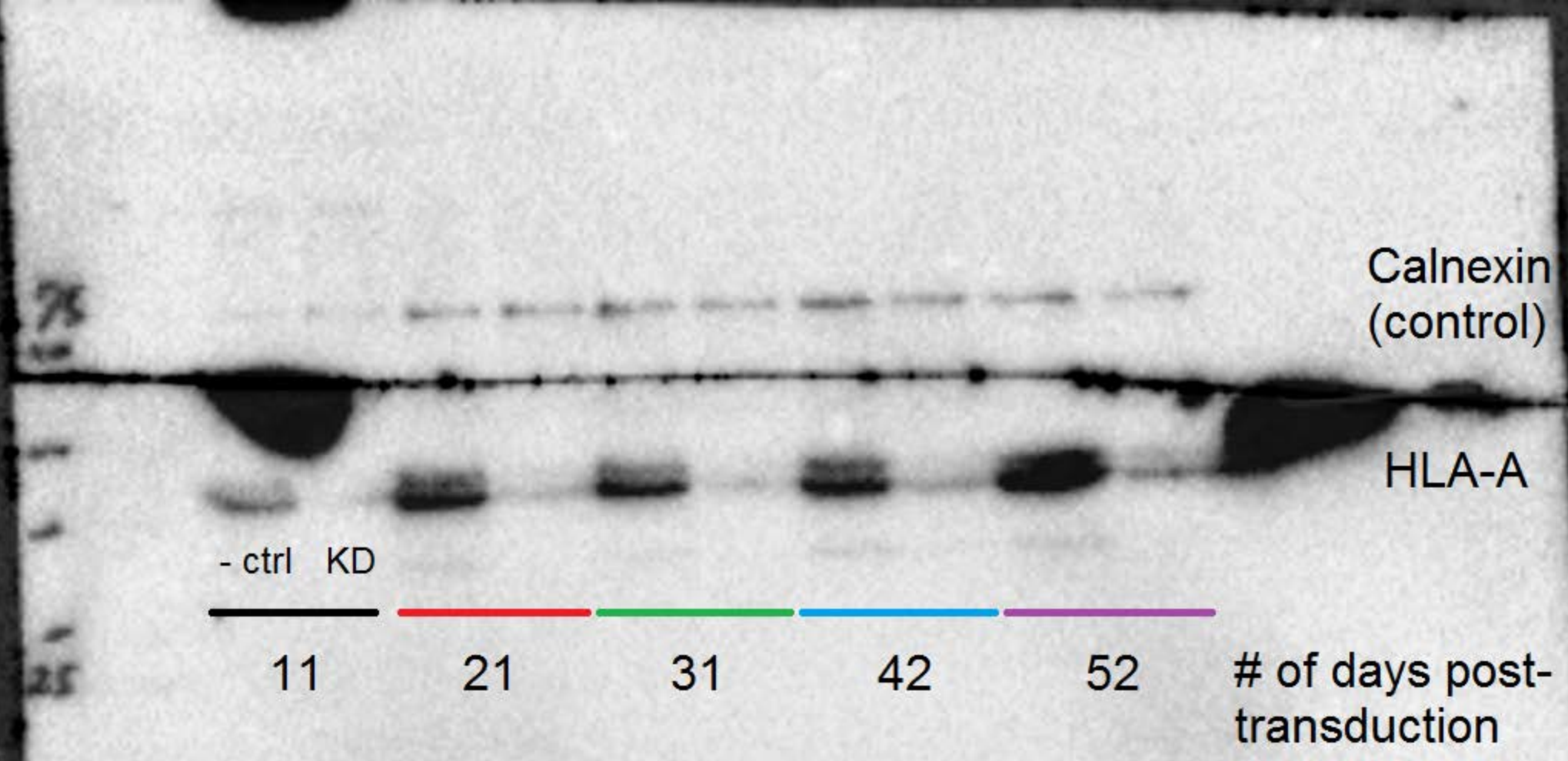

96%

90%

82%

75%

73%

knockdown efficiency  
(normalized intensity of  
HLA-A in knockdown  
divided by neg. control)

Supplement: S1 File — (PDF) [file pone.0332950.s002.pdf]

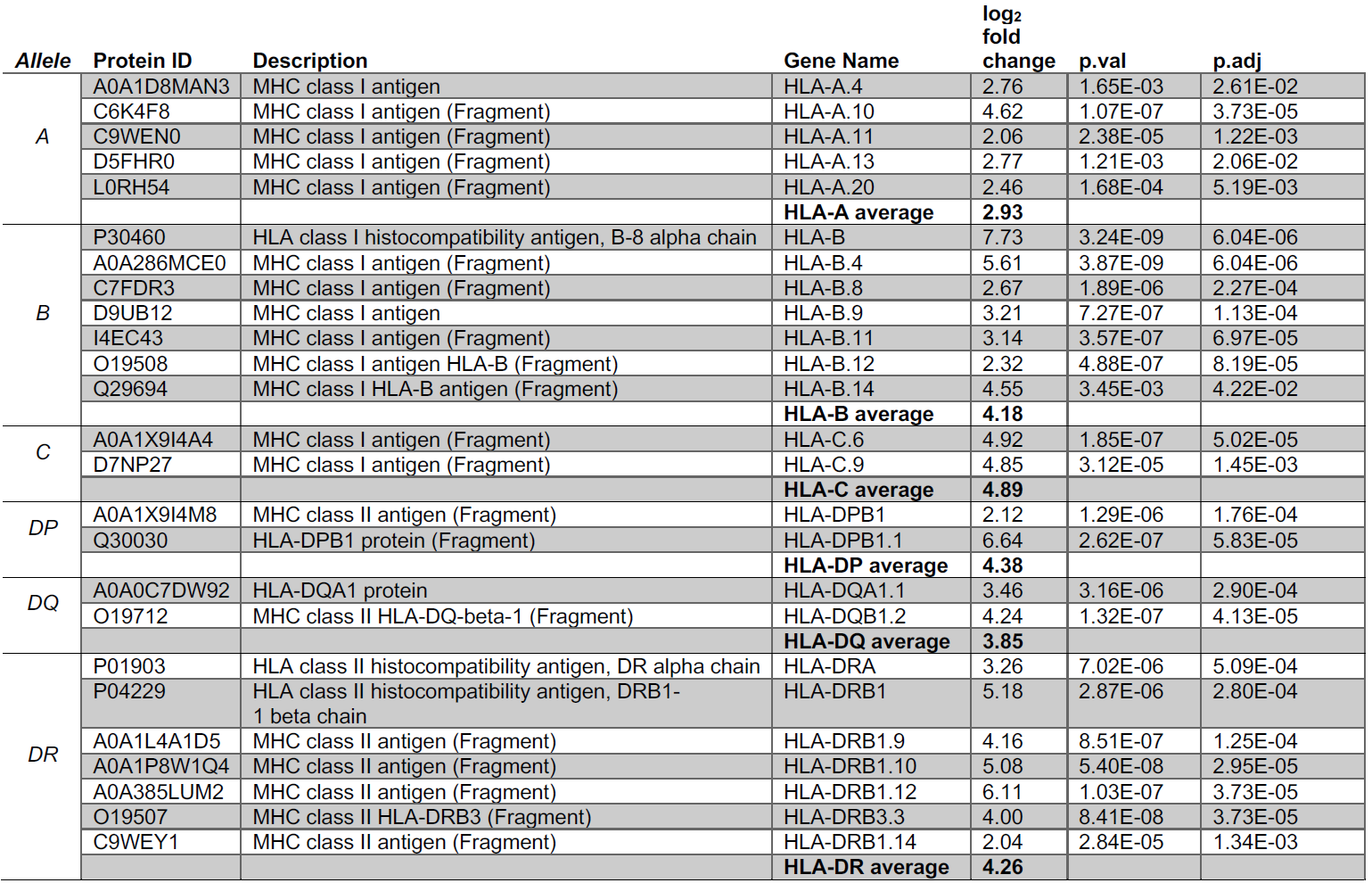

Supplement: S2 Fig — (TIF) [file pone.0332950.s003.tif]

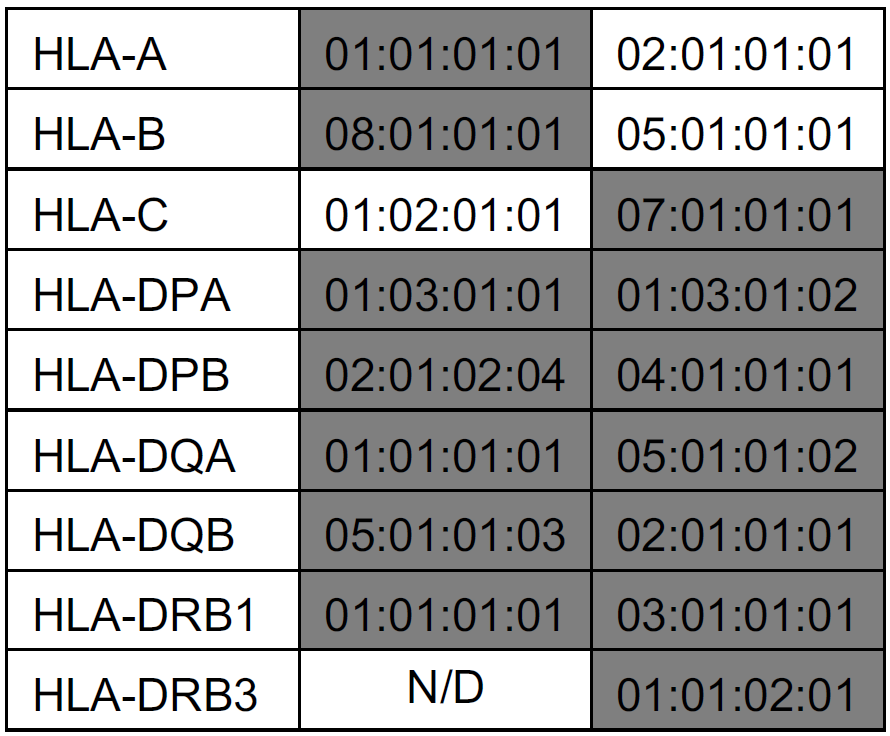

Supplement: S3 Fig — Shaded alleles indicate the absence of detection of that allele in the mutant cells by NGS (N/D = not detected). (TIF) [file pone.0332950.s004.tif]

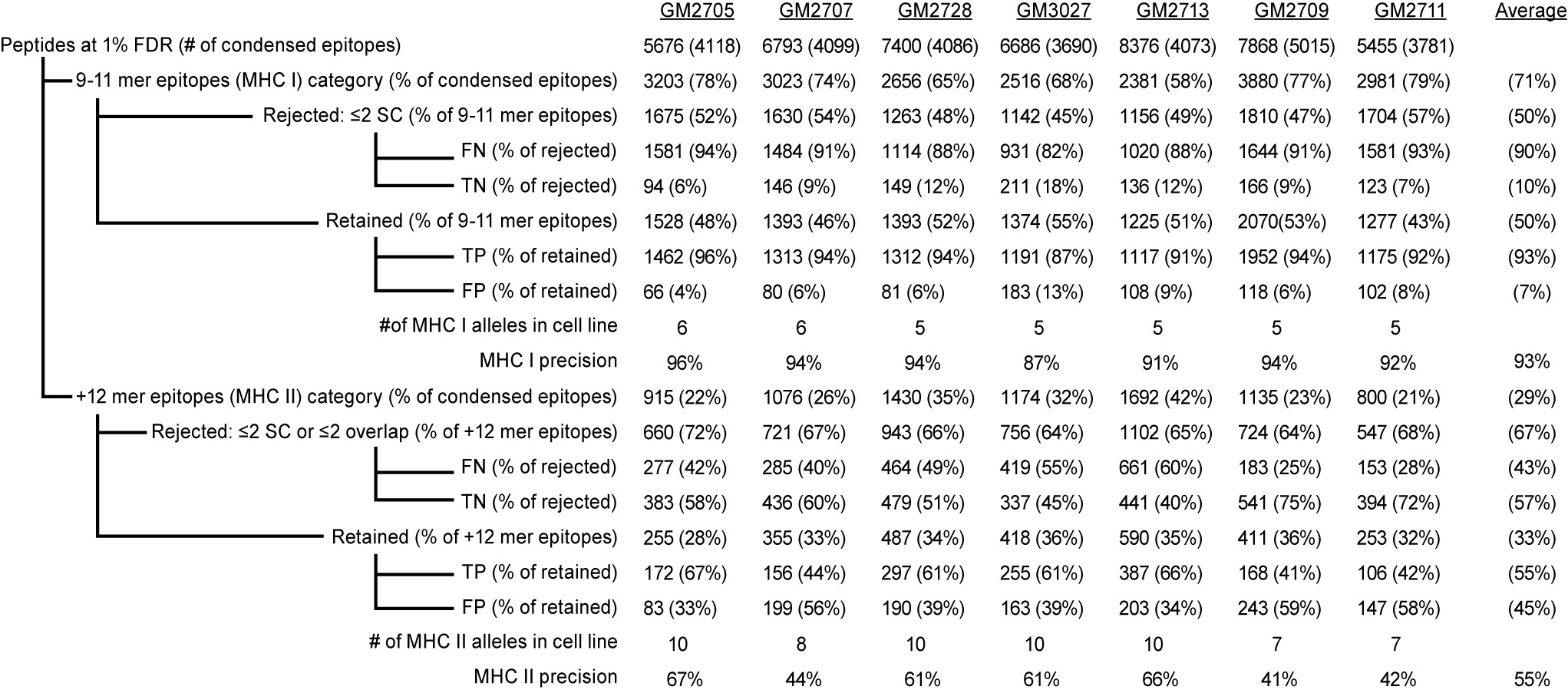

Supplement: S4 Fig — (TIF) [file pone.0332950.s005.tif]

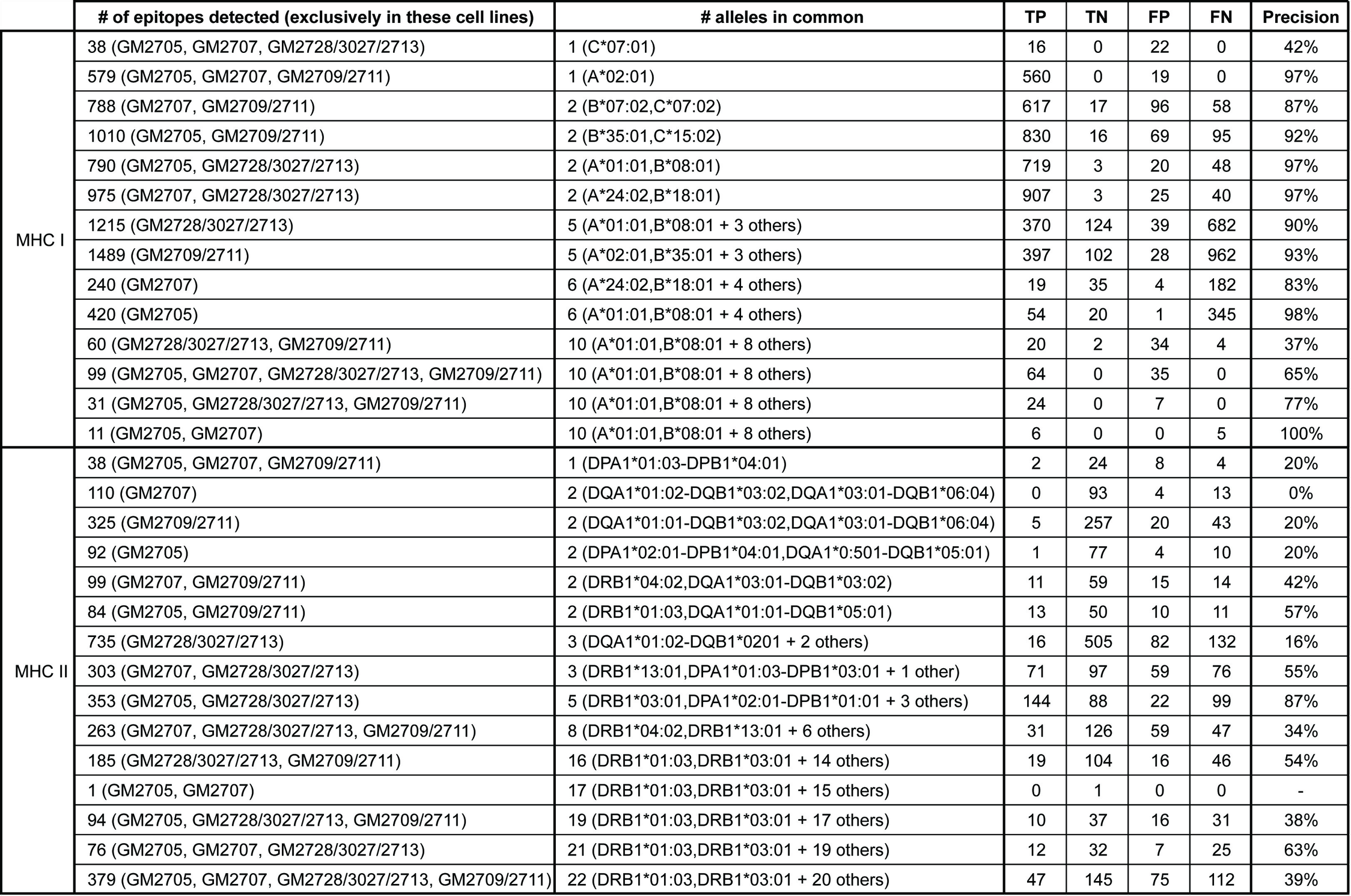

Supplement: S5 Fig — (TIF) [file pone.0332950.s008.tif]

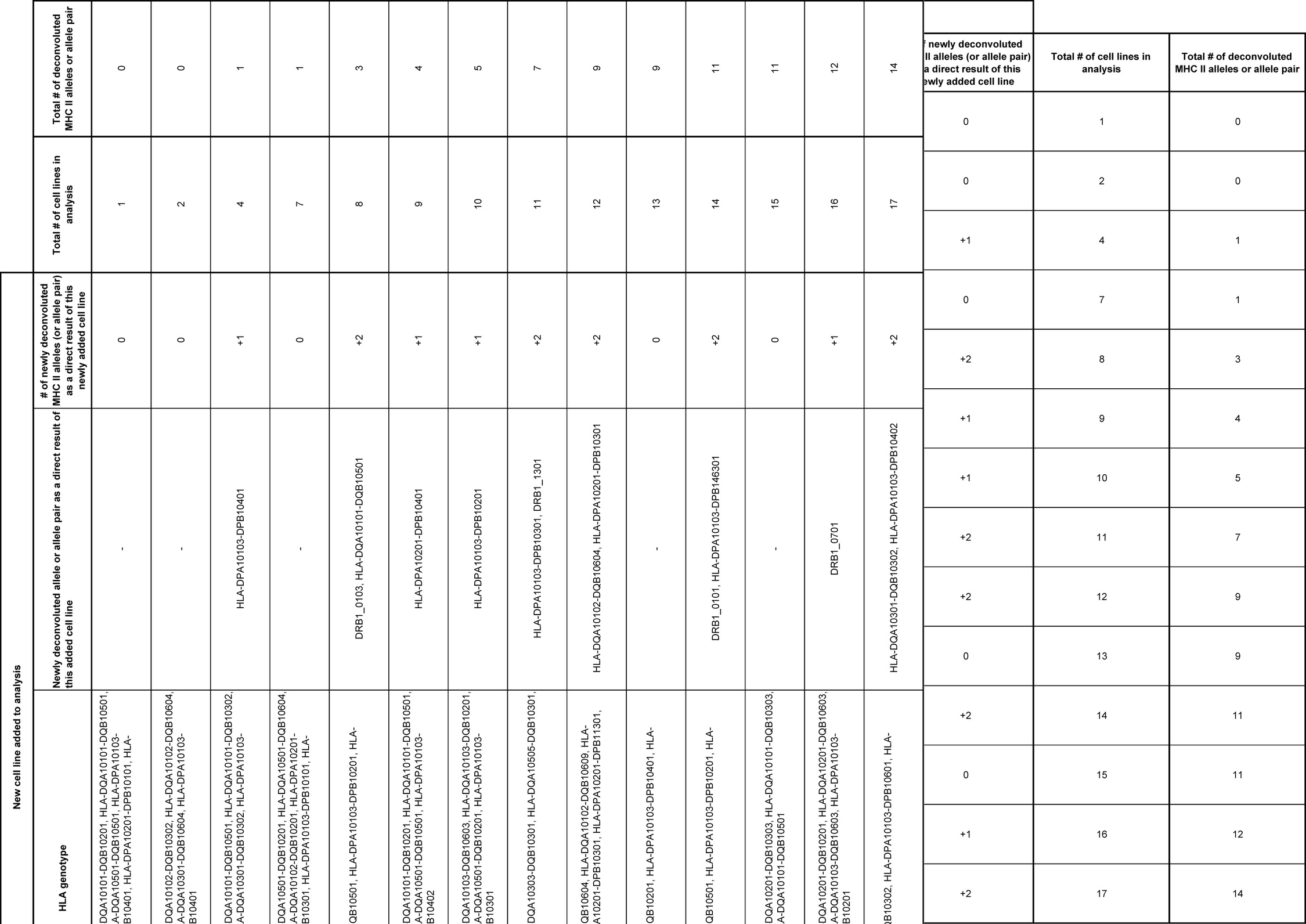

Supplement: S6 Fig — (TIF) [file pone.0332950.s011.tif]
