# Supplementary material for: Framework for analyzing MAE-derived immunopeptidomes from cell lines with shared HLA haplotypes
Source: PLoS One. 2025 Sep 25;20(9):e0332950. doi: 10.1371/journal.pone.0332950 (PMC12463239; doi:10.1371/journal.pone.0332950)
Supplement: Attachment — Submitted filename: PLOS One rebuttal.docx [file pone.0332950.s013.docx]

Response to Editor and reviewers

Response to Editor

I have broken down your comments point-by-point and I address each one directly below each point:

- “The manuscript would benefit from clearer articulation of objectives”
  - Authors’ response:
    - In the Abstract we now state “The goal is to devise an analytical framework to quickly predict immunogenic sequence motifs presented by B-lymphocytes lines from many individuals, akin to recruiting diverse participants in vaccine trials”
    - In the last paragraph of the Introduction we now state “The goal of this project was to develop a methodology for processing MAE-generated data to identify MHC epitopes while minimizing non-specific contaminants, and test how we can take advantage of the genetic connections among the donors to determine which peptides were presented by specific MHC alleles.”
- “The manuscript would benefit from … justification of methodological parameters”
  - Authors’ response: Since this is a methodology paper, justification of the methods we used are made throughout the manuscript, where relevant. Examples of the numerous justifications we now include are:
    - “To avoid “overcounting” highly similar peptides with shared sequences … we coalesced them into epitopes.”
    - “MHC II epitopes must be composed of 3 or more overlapping peptides since this is one of their hallmark traits.”
    - “For MHC I, we limited alignments to a maximum length of 12 residues due to the 9 to 11 residue range of MHC I epitopes.”
    - “For MHC II, we set a maximum alignment length of 30 residues … since their long lengths make it difficult to produce high quality alignments”
- “The manuscript would benefit from … improved statistical validation”
  - Authors’ response: We newly added 2 Results sections that discuss how we benchmarked our data using ligand prediction algorithms. This enabled us to label each epitope we identified as a true positive, false negative, false positive, or true negative. This allowed us to measure the error rate of our methodology. These two new sections are:
    - “Benchmarking epitope sequences derived from peptide data”
    - “Evaluating the impact of epitope clustering and MSA on MHC I and II ligand precision”
- “Discussion could be strengthened by addressing the limitations of MAE, especially for MHC II ligands”
  - Authors’ response: In the Discussion, we now say that..
    - “… non-specific peptide contamination will always be a major disadvantage in MAE”
    - “[MAE] specificity towards MHC II epitopes is clearly inferior to MHC I epitopes”
- “Discussion could be strengthened … comparing the method to existing approaches”
  - Authors’ response: In the Discussion, we now discuss other ligand isolation or prediction methods
    - “…immunoprecipitation method … yields a purer sample of MHC ligands [than MAE].”
    - “informatics tools to predict ligands… comes with an innate possibility for error” , “… their accuracy is highly dependent on the quality of the training data taken from epitope databases.”
    - “… ultimate validation is the use of biological assays that test for CD8 T cell activation by measuring cell proliferation or cytokine release”
- “Structural improvements are also needed, such as reducing overlap between results and methods sections”
  - Authors’ response: Since this is a methodology paper, it is sometimes difficult to decide whether certain procedures should be mentioned in the Methods or Results. Nevertheless, the Methods section has been given a major overhaul to make it much more succinct. Repetitive mentions of certain analytical steps have been moved out of Results and into Methods where possible. Care has been taken not to re-mention of identical procedures.

Response to Reviewer #1

Thank you very much for the constructive criticism. Thank you for being so direct with the improvements you would like to see. I have taken your feedback and put a number to each question/criticism so that I can address each one clearly.

**Q1 from REVIEWER 1:** “the title is informative but slightly long. It could be made more concise while retaining clarity. For example, "Mild Acid Elution-Based Immunopeptidomics for Population-Specific Vaccine Development" might be a more direct option.”

Authors’ response: We agree about the length of the title. It is now shortened to “Framework for analyzing MAE-derived immunopeptidomes from cell lines with shared HLA haplotypes”

**Q2 from REVIEWER 1:** “the methodology could be described more concisely”

Authors’ response: The Methods section has now been almost completely revamped with more headings and subheadings to improve organization and readability. Double mentions of procedures have been eliminated. We placed most of them where they belong – in the Methods. However, given that this is a methodology paper, some procedures that warrant more explaining or justification are in Results.

**Q3 from REVIEWER 1:** “The methodology section provides a detailed protocol, but could you please explain the justification for key parameters such as peptide identification thresholds and FDR values?”

Authors’ response:

- Regarding FDR, we now say “…1% FDR cut off was used at the protein level and peptide level, the acceptable standard used in mass spectrometry”
- Regarding peptide identification threshold, we now state “Peptides found in at least 2 of 3 biological replicates were considered identified to produce a confident and reproducible dataset”
- Regarding spectral count, we now say " To remove sequences with low technical confidence, all epitopes … with a total spectral count of 2 or fewer were removed”
- Regarding the requirement for MHC II epitopes to be made up of at least 3 overlapping peptides, we now state “MHC II epitopes must be composed of three or more overlapping peptides since this is one of their hallmark traits.”

**Q4 from REVIEWER 1**: “While the LC-MS workflow is well described, could you provide more context on how instrument settings were optimized for peptide recovery, particularly regarding the injection volumes for different peptide concentrations?”

Authors’ response: Hope these new sentences address these issues…

- “To maximize peptide identification, fragmentation was allowed for ions of +1 charge (default settings exclude +1), since the peptides were not digested by trypsin and therefore does not always have a +2 charge in an acidic environment.”
- “Peptides were diluted to 1 μg/μL for a 2 μL injection in MS analysis.”

**Q5 from REVIEWER 1**: “Additionally, how do you account for the potential impact of proteolytic cleavage and peptide stability on the observed results, as these factors could introduce confounding variables?”

Authors’ response: We address this issue with these new sentence…

- “Note that it is impossible to differentiate between peptide degradation due to sample handling and natural antigen processing by the immunoproteasome or cathepsins”
- “To avoid “overcounting” highly similar peptides with shared sequences (produced by immunoproteasomes or antigen processing cathepsins, or as a result of sample handling), we coalesced them into epitopes. This avoids the illusion that a sample contains many specific ligands when in fact, many of them are just cleavage products of a larger ligand.”

**Q6 from REVIEWER 1**: “The results section presents strong evidence for the specificity of MAE in isolating MHC I peptides, particularly through HLA-A*02 knockdown validation, but could you clarify why the specificity for MHC II peptides appears lower?”

Authors’ response: We now address this with “…we were surprised to detect 218 epitopes that account for almost 50% of all sequences in the MHC II group, implying that about half of MAE-derived MHC II peptides are non-specific contaminants. This may be because longer peptides tend to be more hydrophobic, therefore more easily adsorb to any surface.”

**Q7 from REVIEWER 1**: Given that a significant proportion of identified peptides may not be true MHC ligands, what steps were taken to verify their relevance?

Authors’ response: To address this concern, a new section has been written (“Benchmarking epitope sequences derived from peptide data“). In summary, we discussed how we used the algorithms NetMHCpan and NetMHCIIpan to benchmark whether the identified peptides are true MHC ligands.

**Q8 from REVIEWER 1**: Additionally, how do you control for the potential contamination of non-MHC peptides, especially in the case of MHC II ligands?

Authors’ response:

In the new section titled “Evaluating the impact of epitope clustering and MSA on MHC I and II ligand precision,” we mention how extra data processing steps (clustering, MSA) can help control or reduce the amount of false positive (contaminant) peptides

“… [MHC II] epitopes deconvoluted to five or fewer alleles and searched against at least one of the well-studied HLA-DRB1 molecules demonstrated precision values ranging from 42% to 87%, with an average of 60%. This represents some improvement compared to the average precision of 55% from cells analyzed independently, without hierarchical clustering and MSA.”

Finally, we acknowledge there might be no feasible way to control for the MHC II contamination problem in any meaningful way “This propensity for [MHC II’s] promiscuous binding implies that there may be no real way to limit contamination in MHC II MAE isolates.”

**Q9 from REVIEWER 1**: To improve reproducibility, could you include a clearer justification for analytical choices, incorporate statistical validation, and explicitly acknowledge the limitations of MAE in detecting MHC II peptides?

Authors’ response:

- We provide more clear justification now for various analytical choices and data filters, such as
  - “…1% FDR cut off was used at the protein level and peptide level, the acceptable standard used in mass spectrometry”
  - “Peptides found in at least 2 of 3 biological replicates were considered identified to produce a confident and reproducible dataset”
  - " To remove sequences with low technical confidence, all epitopes … with a total spectral count of 2 or fewer were removed”
  - “MHC II epitopes must be composed of three or more overlapping peptides since this is one of their hallmark traits.”
  - “For MHC II, we set a maximum alignment length of 30 residues … since their long lengths make it difficult to produce high quality alignments”
- We were able to add some validation statistics once we benchmarked our sequences with NetMHCpan and NetMHCIIpan. All this is discussed under the new sections “Benchmarking epitope sequences derived from peptide data” and “Evaluating the impact of epitope clustering and MSA on MHC I and II ligand precision”
- We explicitly acknowledge the limitations of MAE in detecting MHC II peptides with these newly added phrases
  - “…[MAE] specificity towards MHC II epitopes is clearly inferior to MHC I epitopes”
  - “This propensity for [MHC II’s] promiscuous binding implies that there may be no real way to limit contamination in MHC II MAE isolates”
  - “Admittedly, non-specific peptide contamination will always be a major disadvantage in MAE … especially for MHC II ligands…”

**Q10 from REVIEWER 1**: The introduction of Peptide Index (Pi) and Epitope Index (Ei) is an interesting addition, but could you provide statistical support for these indices, such as variance measures or confidence intervals?

Authors’ response: The idea of using Pi and Ei as an alternative to traditional epitope mapping was indeed very intriguing to us, but we agree with your question/criticism regarding the lack of statistical support for this. As such, the section titled “MAE can be used to approximate epitope mapping for MHC II ligands” that mainly discusses the use of Pi and Ei have now been removed. As for the remaining instances where we use Pi and Ei, they are really just convenient terms for when we average spectral counts. Spectral counting is already a well-established means to quantify peptides and proteins (<https://doi.org/10.1074/mcp.M800203-MCP200>) so I am not sure if I can provide any statistical support for their use.

**Q11 from REVIEWER 1:** To strengthen the results, could you incorporate statistical tests for peptide abundance comparisons, include specificity controls for MHC II peptides, and temper claims about the scalability of the approach?

Authors’ response: I think your question is referring to this paragraph that we have now entirely removed from manuscript:

“MAE can even detect the presence of different epitopes presented by different cell lines within a protein (example in **Fig 2B**). Given that individual peptides within a nested set define an epitope, these individual P_i_ values can also be added together to form a new value, the Epitope Index or E_i_ to approximate an epitope’s abundance relative to other epitopes for a given cell line or compared to other cell lines. Through this method, and removing those with 2 or fewer overlapping peptides, we were able to discover 590 MHC II epitopes presented by the GM2713 cell line.”

This text is now removed because we agree with you that claims about peptide abundance should be back up by statistical tests. In this latest submitted version of the manuscript, the only claim we make about relative peptide abundance is based on spectral count, which is already a widely accepted value that approximates abundance.

Regarding the part of your question about “specificity controls for MHC II peptides”, we hope we have addressed this adequately in our response to your “**Q8 from REVIEWER 1”** (Additionally, how do you control for the potential contamination of non-MHC peptides, especially in the case of MHC II ligands?)

Lastly, regarding the part of your question about “temper claims about the scalability of the approach?” – as mentioned above, that entire paragraph where data from only 1 cell line was used as an example of our experimental/analytical approach has now been removed. Instead, the new section that has been written (“Benchmarking epitope sequences derived from peptide data”) greatly expands the analysis from 1 cell line to 7, with far more detail discussion pertaining to justification of each step, as well as potential error. We hope this helps to address your concerns about scalability of our approach. Broader concerns about the approach’s overall scalability and applicability to far more cell lines (100s?) are addressed in some of your later comments.

**Q12 from REVIEWER 1:** Given that the sample selection is based on a single family, could you address the potential for genetic bias and discuss how this might affect the broader applicability of the method?

Authors’ response: We have now added in the Discussion “the work here is only demonstrated through a single family of seven donor cell lines, and their genetic bias with highly overlapping HLA genotypes among its members rather limits our methodology’s ability to deconvolute MAE-derived epitopes back to their originating MHC. A better approach would be to use cell lines that share some alleles but fewer than that among parents and children. Investigating cell lines sampled from a defined ethnic group should fit the purpose, since they tend to express some alleles in common while maintaining a degree of genetic diversity.”

**Q13 from REVIEWER 1:** The claim that the epitope clustering approach can be applied to large population studies is intriguing, but could you explain how this conclusion is drawn from a dataset of only seven related individuals? Would it be possible to include a discussion on how this technique could be externally validated in more genetically diverse populations? A more balanced discussion should address these points to provide a clearer perspective on the method’s strengths and limitations.

Authors’ response: Continuing along the previous answer, we now added “To illustrate how this can be highly effective, using our approach on merely 10 more cell lines to supplement the existing seven-donor family data will enable the epitopes to theoretically be traced back to an additional 13 MHC II alleles, up from just one from investigating exclusively the seven consanguineous cells. Expanding our research to a much larger population by sourcing hundreds of cells from the 1000 Genomes Project[36] is not merely an aspiration but is already underway at our laboratory.”

**Q14 from REVIEWER 1:** The discussion effectively highlights the potential applications of MAE-based immunopeptidomics for vaccine design, but could you reconsider the generalizability of the findings?

Authors’ response: We concede that “The ultimate validation [of MAE-based immunopeptidomics] is the use of biological assays that test for CD8 T cell activation by measuring cell proliferation or cytokine release. These tests are critical to linking any immunopeptidomics-based ligand discovery methodology with vaccine design… our approach can only be regarded as a ligand and binding motif prediction tool at this time.”

**Q15 from REVIEWER 1:** While predicting allele-specific binding motifs is an important advancement, could you clarify whether these epitopes have been validated for their immunogenicity? Without functional validation, how can their relevance for vaccine development be confirmed?

Authors’ response: We now clearly state “Epitopes that we identified here in this manuscript have not been validated by functional tests for their immunogenicity, and as such, our approach can only be regarded as a ligand and binding motif prediction tool at this time.” With these words, we have taken care not to oversell our methodology’s relevance for vaccine development.

**Q16 from REVIEWER 1:** Additionally, could you provide a more critical evaluation of MAE’s limitations, including potential contamination, scalability challenges, and the method’s reduced specificity for MHC II ligands?

Authors’ response: We have added the following phrases as criticism against MAE

- “… non-specific peptide contamination will always be a major disadvantage in MAE”
- “[MAE’s] specificity towards MHC II epitopes is clearly inferior to MHC I epitopes”
- “This propensity for [MHC II’s] promiscuous binding implies that there may be no real way to limit contamination in MHC II MAE isolates.”
- “…immunoprecipitation method … yields a purer sample of MHC ligands [than MAE].”

**Q17 from REVIEWER 1:** Given that benchmarking against immunoprecipitation-based approaches is missing, could you explain how MAE’s performance compares to existing methods?

Authors’ response: We now acknowledge that benchmarking against MHC IP data better than benchmarking using ligand prediction algorithms (“the immunoprecipitation method, which yields a purer sample of MHC ligands, is a better way to validate MAE-derived epitopes.”) As for comparisons against other techniques, we now discuss other ligand isolation or prediction methods

- “…immunoprecipitation method … yields a purer sample of MHC ligands [than MAE].”
- “informatics tools to predict ligands… comes with an innate possibility for error” , “… their accuracy is highly dependent on the quality of the training data taken from epitope databases.”
- “… ultimate validation is the use of biological assays that test for CD8 T cell activation by measuring cell proliferation or cytokine release”

Response to Reviewer #2

Reviewer #2’s feedback:

“*The article is informative, but the presentation of results appears rushed, lacking depth and clarity. The language needs refinement for accuracy, coherence, and readability, with better word choices and sentence structures. The organization and formatting do not align well with journal standards, requiring improvements in text sequence, size, and adherence to guidelines. Figures should be of higher resolution with clearer interpretations. Substantial revisions are necessary to enhance clarity, structure, and compliance with journal requirements. The authors should address these issues before resubmitting the manuscript*.”

Authors’ response: Thank you for providing some general guidance on how this article can be improved. In this manuscript resubmission, I roughly estimate that 60-70% of the article has been reworked. The data and concepts discussed have not changed, but mostly it has been rewritten to improve readability with better word choice and sentence structure. Major changes include a complete overhaul of the Methods sections, complete with many more headings and subheadings to organize the information. Double-mentions of certain experimental or analytical procedures have been eliminated. Two new sections have been written to discuss the benchmarking of our results. The tedious and complex data filtering approach are now not only described in the text, but visually summarized in Fig 2A and Fig 4B. They are richly annotated to provide useful contextual information. Figure resolution will be optimized once the manuscript is accepted for publication. To adhere to journal guidelines, citations have been re-formatted, unmodified Western blot figure has been added as a supplementary file.

Response to Reviewer #3

Thank you for your feedback. I will address each of the points you made.

1. It would be nice to structure the abstract.

Authors’ response: The abstract is now split into “Background”, “Results”, and “Conclusions”.

2. The objective/aim of the study was not stated both in the abstract and the main article. In the current form , these are implied and leaves the reader in suspesnse.

Authors’ response:

In the abstract, we now state “The goal of this project was to develop a methodology for processing MAE-generated data to identify MHC epitopes, minimize non-specific contaminants, find binding motifs, and utilize genetic connections among donors to determine which peptides were presented by specific MHC alleles.”

In the Introduction, we now clearly state this project’s goals: “The goal of this project was to develop a methodology for processing MAE-generated data to identify MHC epitopes while minimizing non-specific contaminants, find binding motifs, and test how we can take advantage of the genetic connections among the donors to determine which peptides were presented by specific MHC alleles.”

3. Regarding the data analysis The statement given in the first sentence of the first pagraph ie`` on line web application`` should be referenced.

Authors’ response: Done! The relevant paper (DOI: 10.1021/acs.jproteome.4c00294) has now been referenced.

4.Regarding the results The first pagraph is talking about methods and could fit better in the methods section ,Although the results were presented well,in most sections there was inclusion of methods, sometimes discusion in the results. It would be nice to minise them so that the reusts can clearly stand out. most of the methodology statements should be included in the methodology/data analysis and the discussion statements in the duscussion section..

Authors’ response: The Methods section has undergone major changes. There are now many headings and subheadings to organize the information. Due to this reorganization, the Methods section has also been significantly shortened. We have now removed instances where a procedure or protocol was mentioned twice. Most of these have been moved to the Methods section where they belong. However, because this manuscript is about the development of a new experimental/analytical approach, sometimes it is necessary put certain procedures in the Results so we can provide data to justify certain analytical steps. In the Discussion, we have now focused the writing to be more about broader outlooks on this project and evaluating sources of error in our experiment.

5.The authours discussed their results well. However they did not make deefinate conclusions from their results and nor did they make adefinate recommendations.

Authors’ response: We have now re-written the final paragraph of the Discussion to make a summary of this manuscript, highlighting the major findings of this research.

We end with how our approach may be used more widely to benefit vaccine research.

As for making recommendations (future directions?), we discuss the application of our methodology to more cell lines, and mention that efforts are already underway in our laboratory. (“Expanding our research to a much larger population by sourcing hundreds of cells from the 1000 Genomes Project[36] is not merely an aspiration but is already underway at our laboratory.”)
